# Meeting Review: 2002 O'Reilly Bioinformatics Technology Conference

**DOI:** 10.1002/cfg.170

**Published:** 2002-06

**Authors:** Damian Counsell

**Affiliations:** MRC UK Human Genome Mapping Project Resource Centre Hinxton, Cambridge CB10 1SB UK

## Abstract

At the end of January I travelled to the States to speak at and attend the first O’Reilly Bioinformatics Technology Conference [14]. It was a large, well-organized and diverse meeting with an interesting history. Although the meeting was not a typical academic conference, its style will, I am sure, become more typical of meetings in both biological and computational sciences.

Speakers at the event included prominent bioinformatics researchers such as Ewan Birney, Terry Gaasterland and Lincoln Stein; authors and leaders in the open source programming community like Damian Conway and Nat Torkington; and representatives from several publishing companies including the Nature Publishing Group, Current Science Group and the President of O’Reilly himself, Tim O’Reilly. There were presentations, tutorials, debates, quizzes and even a ‘jam session’ for musical bioinformaticists.


**Comparative and Functional Genomics**
*Comp Funct Genom* 2002; **3**: 264–269.
Published online 2 May 2002 in Wiley InterScience (www.interscience.wiley.com). **DOI:** 10.1002/cfg.170

**Feature**

### Meeting Review: 2002 O'Reilly Bioinformatics Technology Conference

Westin La Paloma Resort, Tucson, Arizona, USA. January 28–31 2002.

Damian Counsell*
*MRC UK Human Genome Mapping Project Resource Centre, Hinxton, Cambridge, CB10 1SB, UK*

**Correspondence to:
MRC UK Human Genome Mapping Project Resource Centre, Hinxton, Cambridge, CB10 1SB, UK.
E-mail:
d.counsell@hgmp.mrc.ac.uk*

Received: 2 April 2002
Accepted: 5 April 2002

### Abstract

**At the end of January I travelled to the States to speak at and attend the first O'Reilly Bioinformatics Technology Conference [14]. It was a large, well-organized and diverse meeting with an interesting history. Although the meeting was not a typical academic conference, its style will, I am sure, become more typical of meetings in both biological and computational sciences.**
**Speakers at the event included prominent bioinformatics researchers such as Ewan Birney, Terry Gaasterland and Lincoln Stein; authors and leaders in the open source programming community like Damian Conway and Nat Torkington; and representatives from several publishing companies including the Nature Publishing Group, Current Science Group and the President of O'Reilly himself, Tim O'Reilly. There were presentations, tutorials, debates, quizzes and even a 'jam session' for musical bioinformaticists.** **Copyright ©2002 John Wiley & Sons, Ltd.**

**Keywords:** **bioinformatics; open source; BLAST; visualization; programming; O'Reilly**

### Introduction

Over 700 biologists, computer scientists, bioinformaticists, hackers, publishers and journalists came (some at great personal expense) to Tucson, Arizona to listen, argue, share and to write computer code. In his introduction to one of the keynotes, **Tim O'Reilly** explained why a computer book company and documentation consultancy had organized a biotechnology conference. Last year O'Reilly published its first bioinformatics text [10]. It is not the best introductory bioinformatics text, but imprint's reputation with the so-called 'open source' community was enough to make it immediately (and temporarily) Amazon's best selling computer book.

Many of O'Reilly's loyal readers would happily refer to themselves as 'hackers', meaning 'independent voluntary programmers' rather than 'computer criminals'. (The cognoscenti use the term 'crackers' to refer to the sort of people who deface Web sites or steal passwords.) Hackers lie at the heart of the enormously successful and growing open source software movement.

Open source hackers write computer code and documentation to solve problems, to learn, and to impress others. They make all of their original human-readable program code available (under copyright) for improvement or modification. If others distribute programs based on the original source they are often required by the copyright-holders to make the source code of these changes available to others in turn, otherwise they are free to do what they will with it. Hackers make a distinction between this meaning of 'free', 'free as in speech', and 'free as in beer'.

This ethos and several open source licences have been adopted by most of the major bioinformatics programming projects including EMBOSS [7], Ensembl [8], BLAST [6] and the those of the Open Bioinformatics Foundation [13]: BioPerl, BioJava, BioPython and so on.

### Curious hackers

Many non-scientists were present at this meeting. Part of the success of O'Reilly's foray into bioinformatics publishing derives from the strong and well-intentioned curiosity of this hacker fraternity

Copyright © 2002 John Wiley & Sons, Ltd.

about the maturing field of bioinformatics. Hackers are particularly annoyed, if not always so well informed, by any restriction being placed on the availability of sequence data. They see genomic data as software and are disturbed by any attempt to 'close its source'. They see parallels between software patents (common in the States, but not possible in Europe) and 'gene patents'.

That actual computer programming was a part of the proceedings is testimony to the meeting's unusual background. The 'Bio Hackathon' at the conference was a successful effort in 'live' software development by various programming groups from the **Open Bioinformatics Foundation**. It began the weekend preceding the week of the meeting and continued in Cape Town, South Africa. I must confess that I turned down the chance to participate in this (in place of Alan Bleasby on behalf of the EMBOSS team). Sadly I'm not the sort of programmer who can write substantial programs in days, but I was happy to accept an invitation to speak on the Bioinformatics.Org [3] track.

### Bioinformatics.Org

**Bioinformatics.Org** is a non-profit academic organization, established in 1998 at the University of Massachusetts Lowell. As well as providing a virtual home for a large number of open source bioinformatics projects it campaigns for freedom of information in the biosciences. It is also interested in bioinformatics education. It was true to the spirit of the event that Bioinformatics.Org took over a lecture theatre for its own one-day track.

Bioinformatics.Org's Executive Director, **Jeff Bizarro**, took the opportunity of the O'Reilly meeting – the Second Annual Meeting of Bioinformatics.Org – to present the organization's 2002 Benjamin Franklin Award to **Michael B. Eisen of the Lawrence Berkeley National Laboratory and the University of California at Berkeley**. This award was doubly appropriate, as Eisen wrote ScanAlyze, Cluster and TreeView, immensely popular software for cluster analysis of microarray data, and is also one of the principal moving forces behind the Public Library of Science [16] movement.

### The organization of the conference

It would be impossible to cover all of the talks in detail. I followed my own personal path through the programme and here I report on some of the presentations that made the biggest impression on me.

Presumably to cater for such a broad range of attendees, the conference was sensibly organized along three concurrent technical/academic tracks and one commercial track. I did, however, hear complaints (compliments?) from delegates that too many interesting events coincided.

The 'Fundamentals' track was aimed at both biological and computational beginners. Though I cannot comment on the biological talks on the 'Analysis' track, it certainly carried most of the more heavily computational talks I heard. Most practically oriented was the 'Discovery' track which also carried a couple of presentations about ethical issues in bioinformatics, issues discussed with vigour both inside and outside sessions.

The presentations were further subdivided by style into keynotes, tutorials (with accompanying bound texts) and other, more informal, gatherings (birds-of-a-feather sessions and community meetings).

### 'BLAST programming' – Thomas Madden

Even the least computer-literate biologist will, knowingly or not, have used a BLAST search tool to find identical or similar sequences in the ever-growing genomic databases. Two of the best presentations I attended centred on this marvellous tool. **Thomas Madden** is one of the core BLAST developers at the **NCBI**. For me his talk justified my journey on its own. I am currently planning a collaborative bioinformatics system in which a standalone BLAST server will play a central part.

This tutorial made clear – albeit in fairly technical terms – that BLAST is more than just the most commonly used bioinformatics program. It has developed into a comprehensive and customisable platform with its own storage and output formats, programming interfaces and architecture. If the audience was anything to go by, there is also an active community of programmers and administrators who not only use BLAST, but also have created new variants of the original code.

### 'Linux clusters for bioinformatics' – Glen Otero

Unlike other, proprietary computer operating systems, running Linux on one hundred PCs costs no

Copyright © 2002 John Wiley & Sons, Ltd.

more than running Linux on one PC. Even more surprising, one copy of Linux might well cost you nothing, if you download a so-called ‘distribution’ – a pre-packaged collection of kernel (the core operating system), installation tools, documentation and software from the Net. It’s usually easier, however, to buy a boxed Linux distribution with CDs and manuals. Even this is cheaper than a typical shrinkwrapped edition of Windows.

Linux was not only built across the Net by remotely collaborating programmers, it is also a network-centric operating system. These two attributes are probably connected. Because Linux combines sensible licensing with fast, refined and integrated network capabilities it is perfect for combining computers locally in parallel via Ethernet. Such ‘clusters’ of cheap, generic machines can pool their resources to solve computational problems that could not be tackled by standalones. The power of desktop processors of the Pentium and Athlon lines improves so rapidly and the machines based on these chips are such commodity items that the price/performance characteristics of these systems can overtake those of slower-developing supercomputer architectures. Linux clusters of these and other processors have become popular with many academics both as tools and as objects of study in themselves. **Glen Otero of Linux Prophet** [15] described the application of this technology to bioinformatics.

This was the second tutorial I attended on the first day and any learning-fatigue I might have been suffering had dispersed at the start of Otero’s enthusiastic and jokey presentation. Offering free software CDs for anyone who could use one of a collection of improbable words in a question, juggling with illuminated balls and mocking both himself and his audience Dr. Otero (‘don’t be put off by the ‘Doctor”) gave an extraordinarily comprehensive introduction to the practicalities of choosing and running a Linux cluster in a biological environment. Otero is a consultant to biological researchers thinking of setting up such ‘discount supercomputers’.

### ‘Open source bioinformatics’ – Ewan Birney

**Ewan Birney** is the young and charismatic leader of the **Ensembl group at the European Bioinformatics Institute**. He has always been an enthusiastic advocate of Open Source, both sharing genomic data and sharing bioinformatics source code. His funny and relaxed presentation chimed well with the feelings of the attendees. He joked about the misunderstandings between biologists and computational scientists (over the meanings of words like ‘vector’, for example). He talked about his own work on the Ensembl project and with the various Open Bioinformatics Foundation Open Source projects: BioPerl, BioJava, BioPython, BioCorba, BioDAS.

He also made ‘the case for bioinformatics’. While bioinformaticians are often frustrated by the reluctance of the biological establishment to embrace computational tools for biology, his belief that ‘bioinformatics still hasn’t been hyped enough’ caused me a certain amount of discomfort. Ten years ago, however, people were equally skeptical about the Human Genome project and the prospects for the cloning of large mammals; I hope to be proved a pessimist about the potential of the field.

### ‘Interactive data visualization’ – John Hotchkiss

The original speaker planned for this presentation was the founder of AnVil Informatics, Inc. (AVI), **Georges Grinstein of the University of Massachusetts at Lowell**. Unfortunately he lost his voice before he could talk, but **John Hotchkiss, Chief Technology Officer of AnVil Informatics**, bravely stepped forward to give us a brisk tour of a whole range of technologies – doubly brave since he was presenting someone else’s slides. He did an excellent job.

Genomics produces vast quantities of data. Humans are notoriously poor at absorbing and retaining individual items of information, but famously good at identifying patterns. Hotchkiss began with John Snow’s simple street map plotting of cholera deaths around a water pump in London. The Broad Street pump cholera outbreak of 1854 is now an exemplar in epidemiology. Hotchkiss pointed out that, contrary to the popular image, this map was more a tool of argument than one of investigation. It still makes a convincing case today. This important distinction led to the argument that visualization approaches could usefully be divided according to the kind of interaction users made with the data being processed. Some applications of visualization were for exploratory, some for confirmatory and some for production purposes.

Copyright © 2002 John Wiley & Sons, Ltd. 

As he proceeded, Hotchkiss introduced some of the interesting techniques developed inside and outside his company to handle and clarify multi-dimensional data of the kind commonly encountered in genomics. Some of these techniques were strikingly simple and effective. He showed, for example, that substituting complex icons for coloured dots could separate different populations of data points in plots even more clearly. One of AVI's own proprietary approaches, RadViz, simply maps values onto radii of a circle, but, more cleverly, maximizes the usefulness of this approach by optimising the arrangement of those spokes for revealing patterns.

### 'Data visualization for genomics' – Timothy M. Kunau

**Timothy Kunau of the University of Minnesota** pursued some of these themes further, although his focus initially was more on tools for development such as 'Integrated SYStem' (ISYS), a set of components developed by the National Center for Genome Resources (NCGR) for the exploration of genomic data. ISYS is based on the established Java/Swing programming toolkit. This offered, for example, a metabolic pathway viewer. ISYS seemed to be a perfect example of a tool designed with sharing in mind. Different developers in different labs could produce components independently, but those components had a similar look and feel.

Kunau outlined the 'programming by cartoon' approach of University of Pennsylvania's bioWidgets [5] toolkit. Users can build their own bioinformatics pipeline by assembling visual representations of various modules. For example multiple simultaneous comparisons could be performed by connecting a single sequence input to various processing modules.

He also discussed the visualization techniques used in MetaFam [12], an interfamily protein browser to represent not just the existence of connections between various proteins, but the strengths and nature of those relations.

Finally Kunau addressed the simple practical issues of screen dimensions. You can see more if your screen is bigger. You can see more if you can see in three dimensions. He showed (two-dimensional) slides of a system called 'GeoWall' in action. Based on technology originating at the Electronic Visualization Laboratory of the University of Illinois at Chicago, the GeoWall [9] consortium, including members from the Universities of Minnesota and Michigan, have developed a means of using PCs or Macs to put three dimensions of data onto lecture theatre viewing screens. The system was built with visualization of geological data in mind, but applications to bioinformatics were discussed.

### 'Speedup at what cost?: Heuristic vs. complete algorithms in homology search' – Christopher Dwan

This was one of those rare computational talks aimed at the biological contingent and an outstanding example of the art of explanation. It inspired me to revise my own approach to teaching bioinformatics. **Christopher Dwan of University of Minnesota's Center for Computational Genomics and Bioinformatics** gave the clearest explanation of distinctions and choices to be made in practical bioinformatics (and applied computing in general) that I have ever seen.

He did two difficult things well: explained the specific functional differences between two of the most important sequence alignment algorithms and their consequences for practising researchers, and explained the general distinctions computer scientists make between different classes of problem-solving algorithm.

In this case the two algorithms compared were the complete Smith-Waterman and heuristic BLAST sequence search (alignment) methods. The simple conclusion of this talk was that BLAST is quicker/cheaper and misses some matches while Smith Waterman is slower/more expensive and finds all matches (at least all those possible given a specific scoring system and cut-off). Not only is this a gross over-simplification of Dwan's presentation, but it does no justice to the elegant way Dwan combined empirical demonstration – using data obtained during the conference itself with an exhibitor's system – with good, old-fashioned exposition.

### 'Computing Strategies for the interpretation of mass spectral data for proteomics applications' – William Gleason

**William Gleason** [11] (**University of Minnesota**) gave a witty and insightful talk about some of the most

Copyright © 2002 John Wiley & Sons, Ltd. 

advanced proteomics methods. As biologists drown in nucleotide sequences he emphasised the importance of proteomics to biology and quoted Greg Petsko in describing proteomics as 'going from sequence to consequence'.

With the advent of what he referred to as 'soft' fragmentation techniques such as MALDI (Matrix Assisted Laser Desorption Ionization) and ESI (ElectroSpray Ionization) it has become possible to break proteins up into analysable ions with much less damage. If a technique such as ESI is coupled to capillary electrophoresis or high-pressure liquid chromatography (HPLC) then sequencing of these fragments can be done very rapidly on tiny (femtomolar) quantities of material.

Gleason described how a cluster of Linux PCs (the University of Minnesota Supercomputing Institute Netfinity Linux Cluster) running the Lutefisk1900 and CIDentify software packages could be used to rapidly analyse the fragmentary sequence output from experiments such as these. His goal was to obtain answers sufficiently quickly for the settings of the mass spectrometer to be adjusted in order to maximise the usefulness of its output.

### 'Project management at Bioinformatics.Org' – Gary van Domselaar

As well as being on the Executive Committee of **Bioinformatics.Org**, **Gary van Domselaar** administers Bioinformatics.Org's computer systems. He is a bioinformatics scientist at the **Genetics Institute** and a PhD. Candidate in David Wishart's research group in the **Faculty of Pharmacy and Pharmaceutical Sciences in Edmonton**. He gave a revealing talk, both about the logistics of hosting a wide range of biological computing projects distributed around the globe, and about the nature of some of the projects. Even as a regular visitor and contributor to the Bioinformatics.Org site I learned about several features which were new to me.

### 'DHTML and scalar vector graphics in bioinformatics' – Malay Kumar Basu

One of these revelations was a gem of a project created by **Malay Kumar Basu** in 'downtime' from his full-time study. Basu is a graduate student in Molecular Biology in the **Centre for Cellular and Molecular Biology, India**. SeWeR (Sequence analysis Web Resources) [18] is his ingenious and simple Web interface to an array of server-based bioinformatics programs. It allows a naive user to apply database search, sequence analysis and even visualization programs to his or her data in a simple and customisable way.

Basu's striking talk described the philosophy and technology behind the system. SeWeR is a perfect example of some of the extraordinary individual efforts taking place in the open source software development community. He also spoke about some of his more recent projects, including a Perl library for the generation of scalable vector graphics (SVG) and derived modules for visualizing biological data.

### 'Using the NCBI C++ toolkit in the development of the BIND database' – Doron Betel

This talk was oriented more strongly towards programmers than any other I attended, with detailed source code examples displayed throughout. The NCBI toolkit is an extraordinarily wide-ranging collection of C++ code for bioinformatics software development. In its functionality it rivals the libraries underlying the European Molecular Biology Open Software Suite (EMBOSS). It is a testimony to its accessibility that **Doron Betel** produced his BIND database for the manipulation of biochemical pathway data completely independently of the toolkit's authors. He is a graduate student in the **Chris Hogue's Bioinformatics Lab at the Mt. Sinai Hospital Research Institute in Toronto**; as he put it: 'I'm not at the NCBI and I've never even been there'.

While I was impressed by the vast range of functions available in the kit, its code-generation utility (programs which do their own programming will always be popular with programmers), its documentation and its cross-platform nature, I began to be a little daunted by the level of abstraction at which it operates. For example an elaborate HTML page with multiple hierarchical components can be generated in the toolkit by a single 'print' command, but a firm grasp of such structures is necessary to use this kind of power sensibly.

Copyright © 2002 John Wiley & Sons, Ltd.

### 'BioMail: as an example of push technology in bioinformatics' – Dmitry Mozzherin

BioMail [4] is a classic example of a bioinformatics resource which has become a raging success because it is useful, simple and free. **Dmitry Mozzherin** described how his easy-to-use email publication alert system had caught on with individual biologists and superseded expensive commercial services in several university libraries because of its convenience and reliability.

The system is hosted at Bioinformatics.Org.

### Conclusion

The O'Reilly Bioinformatics Conference managed to be both scholarly and lively. I learned a lot; I discovered bioinformatics projects I had not previously encountered and understood more about familiar projects. While the meeting dealt with science and technology, it also had a 'philosophy'.

Computer programming was originally considered something of an academic pursuit. As it became a big business, the previous science-like ethic of sharing code disappeared. The rise of the Net has revitalized this 'collegiate' approach and inspired the open source movement and its fraternity of self-proclaimed hackers. It would be easy to dismiss them as a semi-anarchic rabble if not for the dazzling successes of the products of their collaborations – including the infrastructure of the Net itself. Programs such as Linux, the free operating system, Apache [1], the most popular Web serving software, BIND [2], the code which assigns identities to almost every internet-connected device, and sendmail [17], which handles the vast majority of email, are all open source creations. In bioinformatics, of course, a great deal of open source code was used to map, sequence and assemble the human genome.

Now that biology and computing are converging it is naturally members of this open source community and who are most eager to bring the philosophy of shared enterprise back to the scientific world whence some feel it came. O'Reilly, as court publishers to the hacker nation may have become accidental pioneers of a new kind of scientific gathering. It is likely that future biotechnological meetings will also be more open to intelligent 'outsiders', more concerned with explanation and more fun.

The Meeting Reviews of Comparative and Functional Genomics aim to present a commentary on the topical issues in genomics studies presented at a conference. The Meeting Reviews are invited; they represent personal critical analyses of the current reports and aim at providing implications for future genomics studies.

### References

1. Apache Software Foundation: **http://www.apache.org/**
2. BIND: **http://www.isc.org/products/BIND/**
3. Bioinformatics.Org: **http://bioinformatics.org/**
4. BioMail: **http://bioinformatics.org/biomail/**
5. BioWidgets: **http://www.cbil.upenn.edu/bioWidgets/**
6. BLAST: **http://www.ncbi.nih.gov/BLAST/**
7. European Molecular Biology Open Software Suite 'EMBOSS': **http://www.emboss.org/**
8. Ensembl: **http://www.ensembl.org/**
9. GeoWall: **http://www.geowall.org/**
10. Gibas C, Jambeck P. 2001. *Developing Bioinformatics Computer Skills*. O'Reilly, Sebastopol, California.
11. Gleason W. homepage: **http://www.cbc.umn.edu/~bgleason/**
12. MetaFam: **http://metafam.ahc.umn.edu/**
13. Open Bioinformatics Foundation: **http://open-bio.org/**
14. O'Reilly Bioinformatics Conference: **http://conferences.oreilly.com/biocon/**
15. Linux Prophet: **http://www.linuxprophet.com/**
16. Public Library of Science: **http://publiclibraryofscience.org/**
17. sendmail.org: **http://www.sendmail.org/**
18. SeWeR: **http://bioinformatics.org/sewer/**

Copyright © 2002 John Wiley & Sons, Ltd.

